# Anaerobic Carbon Monoxide Dehydrogenase Diversity in the Homoacetogenic Hindgut Microbial Communities of Lower Termites and the Wood Roach

**DOI:** 10.1371/journal.pone.0019316

**Published:** 2011-04-26

**Authors:** Eric G. Matson, Kasia G. Gora, Jared R. Leadbetter

**Affiliations:** Ronald and Maxine Linde Center for Global Environmental Science, California Institute of Technology, Pasadena, California, United States of America; Laurentian University, Canada

## Abstract

Anaerobic carbon monoxide dehydrogenase (CODH) is a key enzyme in the Wood-Ljungdahl (acetyl-CoA) pathway for acetogenesis performed by homoacetogenic bacteria. Acetate generated by gut bacteria via the acetyl-CoA pathway provides considerable nutrition to wood-feeding dictyopteran insects making CODH important to the obligate mutualism occurring between termites and their hindgut microbiota. To investigate CODH diversity in insect gut communities, we developed the first degenerate primers designed to amplify *cooS* genes, which encode the catalytic (β) subunit of anaerobic CODH enzyme complexes. These primers target over 68 million combinations of potential forward and reverse *cooS* primer-binding sequences. We used the primers to identify *cooS* genes in bacterial isolates from the hindgut of a phylogenetically lower termite and to sample *cooS* diversity present in a variety of insect hindgut microbial communities including those of three phylogenetically-lower termites, *Zootermopsis nevadensis*, *Reticulitermes hesperus*, and *Incisitermes minor*, a wood-feeding cockroach, *Cryptocercus punctulatus*, and an omnivorous cockroach, *Periplaneta americana*. In total, we sequenced and analyzed 151 different *cooS* genes. These genes encode proteins that group within one of three highly divergent CODH phylogenetic clades. Each insect gut community contained CODH variants from all three of these clades. The patterns of CODH diversity in these communities likely reflect differences in enzyme or physiological function, and suggest that a diversity of microbial species participate in homoacetogenesis in these communities.

## Introduction

Anaerobic carbon monoxide dehydrogenase (CODH) enzymes have been the subject of rigorous biochemical, genetic, and structural investigations. Interest in CODH enzymes stems from their potential biotechnological applications, the remarkable and sometimes extreme lifestyles of the organisms that possess them, and their unusual catalytic properties as these enzymes are among only a few known metalloenzymes that use nickel in the catalytic reaction center. CODH enzymes catalyze the reversible reaction CO + H_2_O↔CO_2_ + 2H^+^ + 2e^−^ and are used in several modes of anaerobic metabolism including carboxidotrophy, acetotrophy, methanogenesis, and acetogenesis (reviewed in [1], [2], [3]).

Unlike the similarly-named (though non-homologous) aerobic carbon monoxide dehydrogenase (CoxL), anaerobic CODH is a central enzyme in the Wood-Ljungdahl (acetyl-CoA) pathway for acetogenesis. This metabolism is distributed among only a few phyla but is found in bacteria inhabiting many anaerobic environments, both free-living and host-associated [4], [5]. Acetogenic bacteria use CODH in conjunction with acetyl-CoA synthetase (ACS) to catalyze the reduction of CO_2_ to CO through the carbonyl branch of the acetyl-CoA pathway [6], [7], [8]. This enables acetogenic bacteria to grow chemolithoautotrophically, whereby CO_2_ serves as the carbon source and hydrogen (or H_2_-equivalents) provides the reducing power to drive the metabolism [3]. Via the CODH/ACS enzyme complex, acetogenic bacteria form acetyl-CoA, which can either be used for energy conservation or generation of cell carbon.

Acetogenesis is important to the mutualism occurring between termites and their gut microbiota. Microbial communities in the gut tracts of wood-feeding termites generate large quantities of CO_2_ and H_2_ as byproducts of lignocellulose-derived carbohydrate fermentation [9], [10]. In the termite gut environment, H_2_ can reach standing concentrations that approach saturation and are among the highest levels measured in biological systems [11], [12]. H_2_ and CO_2_ are retained in the gut where acetogenic bacteria convert them into acetate, the primary energy source for the termite. These bacteria contribute up to one third of the total acetate pool in the gut, and therefore comprise an important component of the termite gut community [10], [13], [14].

CODH enzymes are both sequence and functionally diverse [15]. An initial view into the diversity of genes encoding CODH enzymes in termites was provided by the metagenome sequence of the gut microbial community of a phylogenetically-higher termite species belonging to the genus *Nasutitermes* [16]. This dataset was found to contain 2 full-length *cooS* genes, which encode the catalytic (β) subunit of the CODH enzyme complex and ca. 30 short *cooS* gene fragments. The diversity of these fragments suggested that termite gut communities may contain numerous CODH enzyme variants. We therefore sought to identify *cooS* genes in bacterial species isolated from the termite gut and to investigate patterns of *cooS* diversity in other insect gut communities.

Herein, we report the development and application of degenerate PCR primers designed to amplify *cooS* genes. To our knowledge, the primers we developed are the first to target genes encoding anaerobic CODH enzymes in the environment, although primer sets targeting *acsB*, which encodes acetyl-CoA synthetase (the partner enzyme of anaerobic CODH) have recently been reported [17]. Our primers are capable of amplifying a broad diversity of *cooS* genes and thus provide a molecular tool for understanding the genetic and possible functional diversity of carbon monoxide genes in anaerobic environments, both free-living and host-associated.

We used these primers to identify *cooS* genes in termite-gut bacterial isolates belonging to the genus *Treponema* of the phylum *Spirochaetes* and to sample *cooS* diversity in three phylogenetically-lower termite species, *Zootermopsis nevadensis*, *Reticulitermes hesperus*, and *Incisitermes minor*. These termite species represent families *Termopsidae*, *Kalotermitidae* and *Rhinotermitidae*, respectively, which constitute half of all major lines of phylogenetically-lower termite descent [18]. As acetogenesis also occurs in wood-feeding cockroaches (family *Cryptocercidae*) and to some extent in omnivorous cockroaches [13], we also investigated *cooS* diversity in *Cryptocercus punctulatus* and *Periplaneta americana*, representatives of these other dictyopteran insects. The *cooS* genes we identified in bacterial isolates and in insect gut inventories encode variants that are only distantly related to CODH enzymes that have previously been biochemically and/or structurally characterized. We also observed marked differences in the distribution of the *cooS* sequence and potential functional diversity among termites and cockroaches.

## Results

### Design and use of *cooS* modular primer sets

We undertook a modular approach to primer design to retrieve *cooS* genes from environmental sources and overcome challenges presented by the broad sequence diversity of CODH enzymes and the semi-conserved nature of the *cooS* primer binding sites. Sixteen degenerate primers were designed for use as primer modules in a variety of forward and reverse combinations (Table 1). The forward primer modules cooS-1F, cooS-2F, cooS-3F and cooS-4F target all possible combinations of nucleotide sequences encoding semi-conserved amino acid blocks MGPCRID, MGPCRIT, MGPCRIS and NGPCRIT, respectively. This region maps to the 5′ end of *cooS* and includes a cysteine residue that forms part of a conserved [Fe4-S4] metal cluster known as the cubane center or B-cluster [19], [20]. The reverse primer modules cooS-1R, cooS-2R and cooS-3R target all possible combinations of nucleotide sequences encoding semi-conserved amino acid blocks PPV/ILHMG, PPV/ILNFG, and PPV/ILHLG, respectively. This region maps to the 3′ end of the gene adjacent another conserved cysteine that forms part of the Ni-Fe-S center or C-cluster where catalysis occurs [19], [20]. These regions are located approximately 1350 bp apart, a distance that encompasses approximately 70% of the typical *cooS* gene.

**Table 1:** Primer modules for amplifying *cooS*, which encodes the catalytic (β) subunit of anaerobic CODH enzymes.

Forward Primer Module Sequences (5′ - 3′)								Fold Degeneracy	Unique Priming Sequence Targets
**cooS-1F**	ATG	GGI	CCI	TGY	AGR	ATH	GA	18	576
	ATG	GGI	CCI	TGY	CGI	ATH	GA		
**cooS-2F**	ATG	GGI	CCI	TGY	AGR	ATH	AC	18	576
	ATG	GGI	CCI	TGY	CGI	ATH	AC		
**cooS-3F**	ATG	GGI	CCI	TGY	AGR	ATH	TC	36	1152
	ATG	GGI	CCI	TGY	CGI	ATH	TC		
	ATG	GGI	CCI	TGY	AGR	ATH	AG		
	ATG	GGI	CCI	TGY	CGI	ATH	AG		
**cooS-4F**	AAY	GGI	CCI	TGY	AGR	ATH	AC	36	1152
	AAY	GGI	CCI	TGY	CGI	ATH	AC		

Reverse Primer Module Sequences (5′ - 3′)								Fold Degeneracy	Unique Priming Sequence Targets
**cooS-1R**	CCC	ATR	TGI	ARN	ACI	GGI	GG	28	1792
	CCC	ATR	TGI	ARD	ATI	GGI	GG		
**cooS-2R**	CCR	AAR	TTI	ARN	ACI	GGI	GG	56	3584
	CCR	AAR	TTI	ARD	ATI	GGI	GG		
**cooS-3R**	CCI	ARR	TGI	ARN	ACI	GGI	GG	56	14336
	CCI	ARR	TGI	ARD	ATI	GGI	GG		

Cumulatively, the primers are 248-fold degenerate and have the potential to target a total of 68,124,672 unique combinations of forward and reverse primer binding sequences. The primers were determined to include forward and reverse combinations that matched 27% of the *cooS* genes in the GenBank Reference Sequence database of closed and partially sequenced microbial genomes, a result that reflects the large diversity of CODH enzymes. Our primers are designed to target *cooS* genes in a variety of microbial species belonging to phyla *Firmicutes*, *Proteobacteria*, and *Euryarchaeota* and *cooS* genes predicted to belong to *Treponema* sp. in the *Nasutitermes* sp. metagenome [16].

Despite the use of deoxyinosine bases in the primer design, which do not contribute to degeneracy and have low selectivity with respect base pairing stability [21], the primer modules are still highly degenerate. This necessitates the use of large quantities of primer in PCRs. Primer modules produce sufficient PCR products with minimal amplification of non-target sequences when used at a final concentration of 0.2 pmol⋅ul^−1^⋅degeneracy^−1^⋅primer^−1^ (Fig. S1). Non-reacted primers and some non-specific amplicons were removed by gel-purifying the 1.4 kb PCR products.

**Figure 1:**
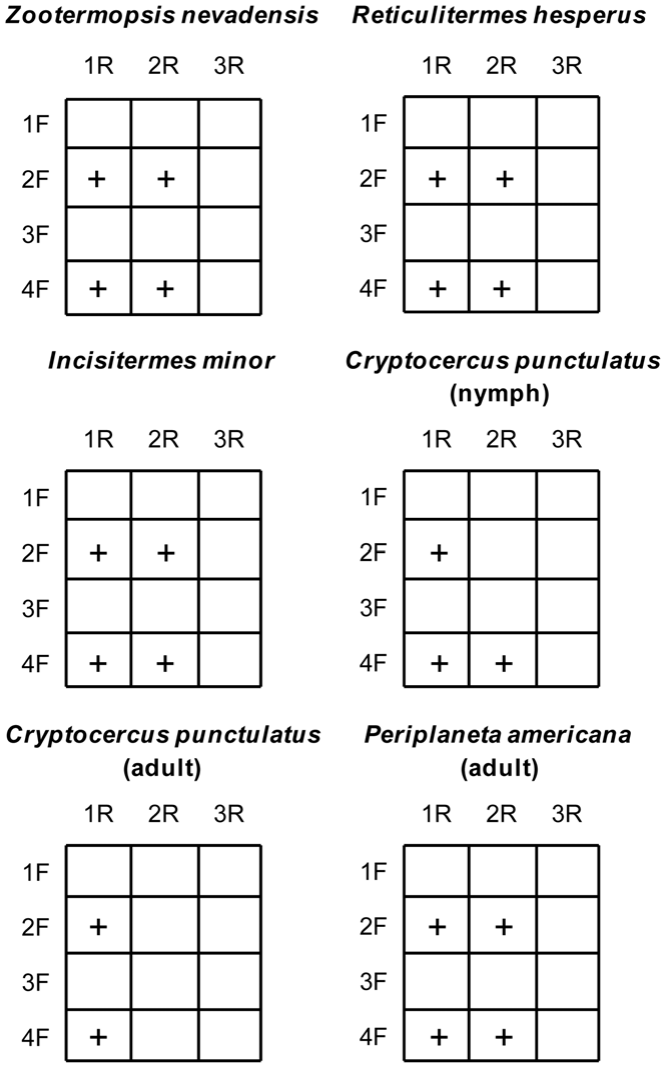
Combinatorial amplification of insect gut community DNA using *cooS* forward and reverse primer modules. 1.4 kb PCR amplicons produced by each forward and reverse primer module combination are denoted by+symbols where detected. No symbol denotes that a 1.4 kb product was not detected for these primer module combinations under our PCR conditions. Abbreviated labels 1F–4F and 1R–3R refer to primer modules cooS-1F–cooS-4F and cooS-1R–cooS-3R, respectively.

Pair-wise PCR was used to determine primer module combinations that produce an amplicon product of the expected size for *cooS* genes (Fig. 1). Using only productive primer modules to generate amplicons for cloning reactions reduced non-specific PCR amplification of community DNA templates. Although primer module cooS-1F, cooS-3F, and cooS-3R did not produce detectable products from any of the templates analyzed in this study, each of these primer modules amplified *cooS* genes from gut communities of phylogenetically-higher termite species in a subsequent study (manuscript in preparation).

### *cooS* inventories

A *cooS* gene was amplified and sequenced from each of six spirochetes belonging to the genus *Treponema* (*T. primitia* str. ZAS-1, *T. primitia* str. ZAS-2, *T. azotonutricium* str. ZAS-9, and *Treponema* sp. ZAS-5, ZAS-6 and ZAS-8), all of which were previously isolated from the gut of the termite *Zootermopsis angusticolis* [22], [23]. The diversity of amplified *cooS* genes was surveyed in *Z. nevadensis*, *C. punctulatus*, and *P. americana* by RFLP analysis of a 95-clone library generated from the gut communities of each of these insects. The number of RFLP types was greatest for the *Z. nevadensis* library, followed by *C. punctulatus*, and finally, *P. americana* (Table S1 and Fig. S2). As a result of non-specific amplification, several of the clones contained non-target DNA and were not further analyzed. To examine whether or not the diversity of *cooS* genes in *R. hesperus* and *I. minor* was similar to *Z. nevadensis*, 24 randomly-selected clones from these other phylogenetically-lower termites were sequenced. An additional 24 randomly-selected clones from an adult specimen of *C. punctulatus* were sequenced to verify *cooS* diversity observed in the nymph. A nymph of *P. americana* was not found at the collection site of this species. In total, 6 *cooS* clones from termite gut bacterial isolates and 145 *cooS* clones from gut microbial communities of termite and cockroach species were sequenced in this study (Table S1). The environmental *cooS* clones were condensed into operational taxonomic units (OTUs) based on 97% translated amino acid similarity, resulting in 85 total OTUs including 27 from *Z. nevadensis*, 13 from *R. hesperus*, 9 from *I. minor*, 17 from *C. punctulatus*, and 19 from *P. americana*.

### Phylogenetic relationships among CooS sequences

**Figure 2:**
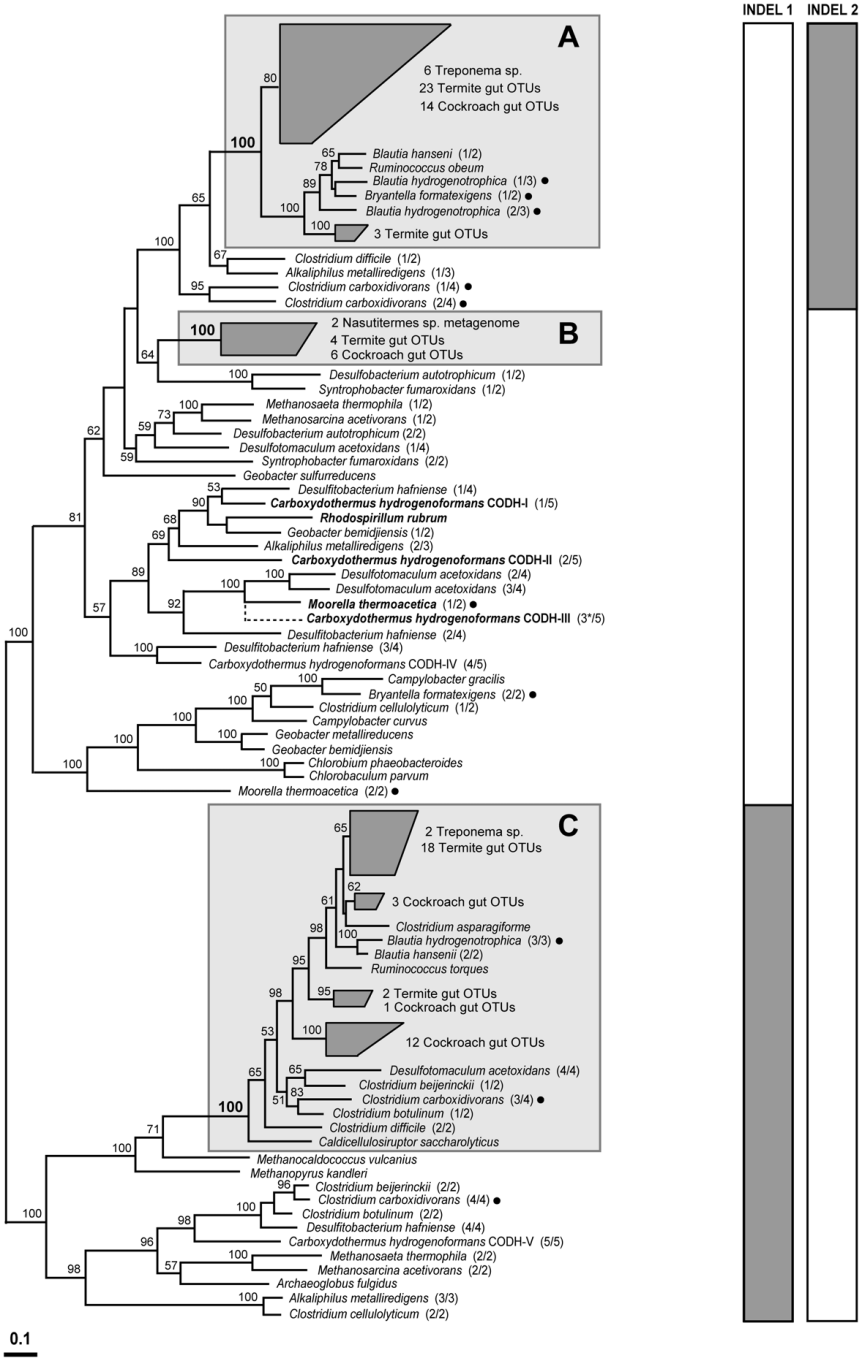
Protein phylogeny and independent character analysis of translated amino acid sequences of *cooS* genes. Protein phylogeny was determined by Phylip ProML maximum-likelihood analysis of 379 unambiguously aligned and filtered amino acid positions. Support for the tree was calculated by 100 bootstrap-step reanalysis of the dataset using protein maximum-likelihood methods and is reported for nodes with greater than 50% support. Gray boxes (labeled A, B, and C) indicate the major clades of CooS sequences determined in this study along with related representatives from described microbial isolates. Clade designations are defined by 100% bootstrap support and branch-length separation from other CooS sequences. Bold labels are CODH enzymes that have been biochemically or structurally characterized. Dots indicate CooS sequences from species shown to be CO_2_-reductive acetogens [4]. For species having more than one CODH, the number of the CODH out of the total is given parenthetically. *The sequence of CODH-III of *C. hydrogenoformans* contains a reading frame shift; the approximate phylogenetic position of its corrected amino acid sequence is given by the dashed line. Bar indicates distance given as 0.1 changes per amino acid position. Side bars indicate the results of independent character analysis of insertion/deletion (indel) sequences in each of the CODH proteins. Indel 1: sequences indicated by the gray bar contain the amino acid sequences TEIFD/NGH corresponding to position 306–312 in *T. azotonutricium* and *T. primitia* str. ZAS-1. Indel 2: sequences indicated by the gray bar contain the amino acid sequences A/TNS/THVD corresponding to position 409–414 in *T. azotonutricium* or 399–404 in *T. primitia* str. ZAS-1 and *T. primitia* str. ZAS-2.

**Figure 3:**
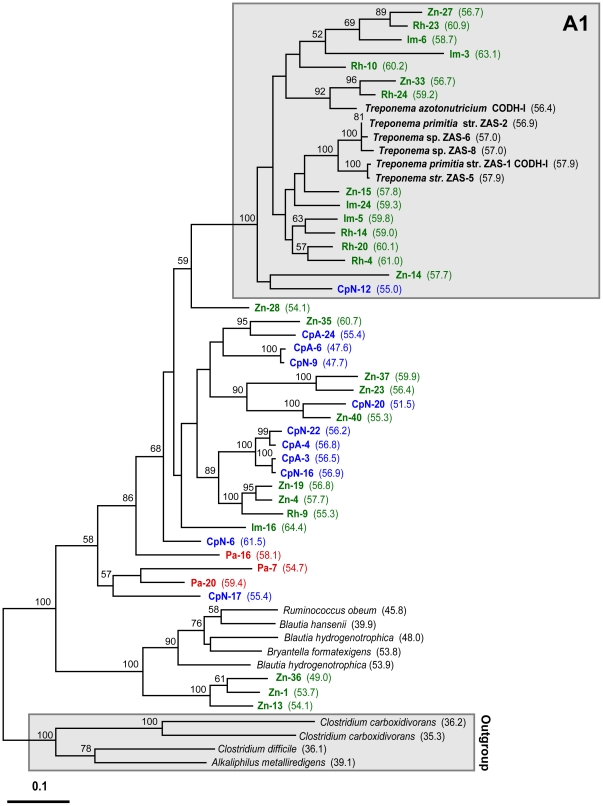
Phylogeny of clade A-associated CooS sequences. Protein phylogeny was determined by Phylip ProML maximum-likelihood analysis of 423 unambiguously aligned and filtered amino acid positions. Support for the tree was calculated by 100 bootstrap-step reanalysis of the dataset using protein maximum-likelihood methods and is reported for nodes with greater than 50% support. Sequences recovered from insect gut inventories are indicated as follows: termite sequences (green) are from *Z. nevadensis*, Zn; *R. hesperus*, Rh; and *I. minor*, Im; wood-feeding cockroach sequences (blue) are from *C. punctulatus* adult, CpA and nymph, CpN; omnivorous cockroach sequences (red) are from *P. americana*, Pa. %G+C content of *cooS* genes for each translated amino acid sequence is given parenthetically. Group A1, indicated by a gray box, contains CooS sequences putatively assigned to the genus *Treponema* based on phylogenetic inference. Bar indicates distance given as 0.1 changes per amino acid position.

**Figure 4:**
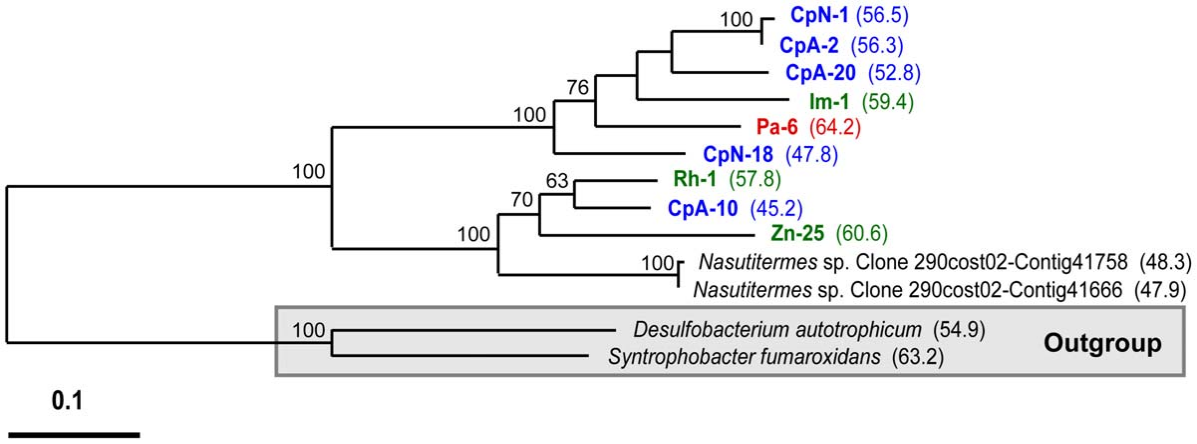
Phylogeny of clade B-associated CooS sequences. Protein phylogeny was determined by Phylip ProML maximum-likelihood analysis of 436 unambiguously aligned and filtered amino acid positions. Support for the tree was calculated by 100 bootstrap-step reanalysis of the dataset using protein maximum-likelihood methods and is reported for nodes with greater than 50% support. Sequences recovered from insect gut inventories are indicated as in figure 3. %G+C content of *cooS* genes for each translated CooS amino acid sequence is given parenthetically. Genes identified in the *Nasutitermes sp.* metagenome are indicated with clone designations as they appear in the Joint Genomes Institute Img/m database. Bar indicates distance given as 0.1 changes per amino acid position.

**Figure 5:**
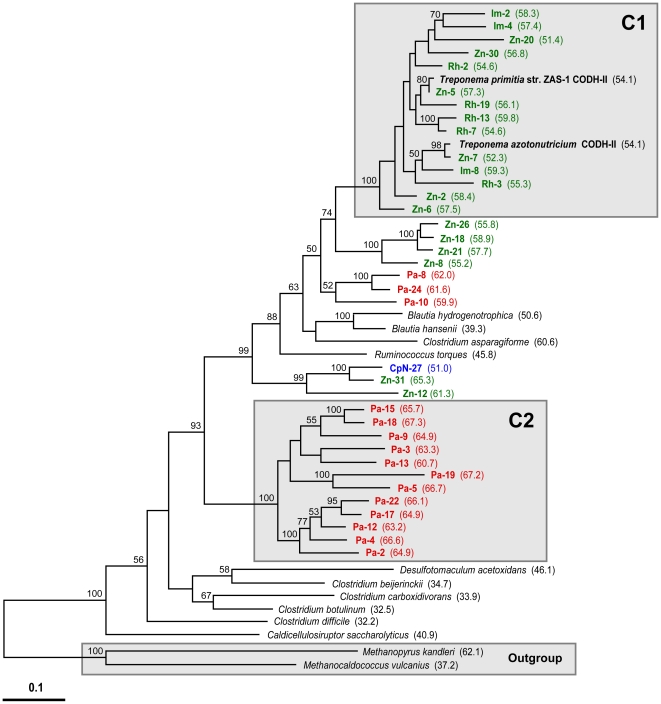
Phylogeny of clade C-associated CooS sequences. Protein phylogeny was determined by Phylip ProML maximum-likelihood analysis of 424 unambiguously aligned and filtered amino acid positions. Support for the tree was calculated by 100 bootstrap-step reanalysis of the dataset using protein maximum-likelihood methods and is reported for nodes with greater than 50% support. Sequences recovered from insect gut inventories are indicated as in figure 3. %G+C content of *cooS* genes for each translated amino acid sequence is given parenthetically. Group C1, indicated by gray box, contain CooS sequences putatively assigned to the genus *Treponema* based on phylogenetic inference. Group C2, indicated by gray box, contains CooS sequences of unknown phylogeny that were found only in the omnivorous cockroach, *P. americana*. Bar indicates distance given as 0.1 changes per amino acid position.

Protein phylogenies of deduced amino acid sequences of *cooS* genes contained in our inventories showed that CODH OTUs all group within one of three distantly-related phylogenetic clades. These clades are separated by long branch-lengths and are supported by robust (100%) bootstrap values (Fig. 2). We calculated similarity matrices using an aligned cumulative dataset containing all of the OTUs together and separately for OTUs belonging to each of the clades. The average amino acid similarity between OTUs belonging to clade A and B is 45% (range 41–50%), between clade B and C is 29% (range 26–32%), and between clade A and C is 27% (range 23–35%). We also observed a large amount of within-clade sequence divergence wherein the two most distantly related OTUs from clade A, B, and C share only 57%, 62%, and 63% amino acid similarity, respectively. Within each of the clades, the %G+C content of *cooS* sequences encoding CODH OTUs also varies widely with values ranging between 47.6%–64.9%, 45.2%–60.6%, and 51.0%–67.3% within clades A, B, and C, respectively (Fig. 3, Fig. 4, Fig. 5).

Clades A, B, and C all contain OTUs from each of the insect gut communities investigated in this study. Sequences grouping within these clades contain different insertion/deletion sequences (indels), although the presence or absence of these features is not limited to the defined clades (Fig. 2, side bars). OTUs in clade A contain a 6 amino acid indel of varying sequence composition (corresponding to A/TNS/THVD located from amino acid position 409–414 in *T. azotonutricium* or 399–404 in *T. primitia* str. ZAS-1 and ZAS-2) while those in clade C contain a different, 7 amino acid indel (corresponding to TEIFD/NGH located from amino acid position 306–312 in *T. azotonutricium* and *T. primitia* str. ZAS-1). OTUs in clade B have gaps at both of the corresponding indel positions in clade A and clade C. As the indels were not considered in the construction of phylogenetic trees, they serve as an independent confirmation of the phylogenetic differences among clades.

Clade A (Fig. 3) contains a deduced amino acid sequence from a *cooS* gene from each of the 6 termite gut *Treponema* sp. These sequences group together except for *T. azotonutricium*, which groups instead with OTUs fom *Z. nevadensis* and *R. hesperus*. Clade A also contains the majority of OTUs from termites *Z. nevadensis*, *R. hesperus*, *I. minor*, and the wood-feeding cockroach *C. punctulatus* (52%, 54%, and 56%, and 65%, respectively) while only three (16%) of the OTUs from the omnivorous cockroach *P. americana* group within this clade. Many of the sequences from termite gut communities that group within clade A form a well-supported subclade (group A1) that also includes a CooS sequence from each of the *Treponema* isolates (Fig. 3). Clade A also contains CooS sequences from clostridial members of the phylum *Firmicutes*. These clostridial sequences genes group together along with a small sister clade comprised of three *Z. nevadensis* OTUs.

Clade B (Fig. 4) contains the least number of OTUs identified in this study and no known CooS sequences from microbial isolates. Only a single OTU each from termites *Z. nevadensis*, *R. hesperus*, *I. minor*, and the omnivorous cockroach *P. americana* group within this clade, while five (29%) of the OTUs from the wood-feeding cockroach *C. punctulatus* group within the clade. Phylogenetic relationships between clade B and other *cooS* genes from microbial species could not be determined due to a lack of closely related homologs in databases and low bootstrap support for nodes below the clade B designation. However, the amino acid sequences of two full-length *cooS* genes from the *Nasutitermes* sp. metagenome group within clade B and were previously predicted to belong to the genus *Treponema* based on bioinformatics analysis [16].

Clade C (Fig. 5) contains a CooS sequence from two of the termite gut spirochete isolates, *T. primitia* str. ZAS-1 and *T. azotonutricium*, both of which have a second CooS grouping within clade A. While the clade A-associated *cooS* genes in both of these species were identified by our primers, our primer sets did not amplify the clade C-associated *cooS* gene in either species, which were later identified by sequencing the genomes of these spirochetes. Thus, although Clade C contains numerous OTUs from termites *Z. nevadensis*, *R. hesperus*, and *I. minor* (44%, 38%, and 33%, respectively), it is the possible that others were not sampled. The majority (79%) of OTUs from the omnivorous cockroach *P. americana* group within clade C. In contrast, only a single OTU from the wood-feeding cockroach *C. punctulatus* groups within this clade. Most of the termite gut OTUs group together with CooS sequences from *T. primitia* str. ZAS-1 and *T. azotonutricium* to the exclusion of those from cockroaches, forming a well-supported subclade (group C1). A second subclade within clade C (group C2), is separated by a long branch-length and also has robust bootstrap support. This subclade is populated exclusively by OTUs from the omnivorous cockroach *P. americana*. As with clade A, in addition to CooS sequences from species of *Treponema*, clade C also contains CooS sequences from bacterial species belonging to the subphylum *Clostridia*.

### *cooS* gene neighborhoods

Termite gut spirochetes *T. primitia* str. ZAS-1 and *T. azotonutricium* contain more than one *cooS* gene, as do many of the other bacterial species analyzed in this study. In an effort to understand possible functional roles of the various anaerobic CODH enzymes, we undertook an analysis of several microbial genomes and the *Nasutitermes* sp. metagenome for gene neighborhood context of *cooS* genes associated with each of the three clades into which our protein sequences group.

**Figure 6:**
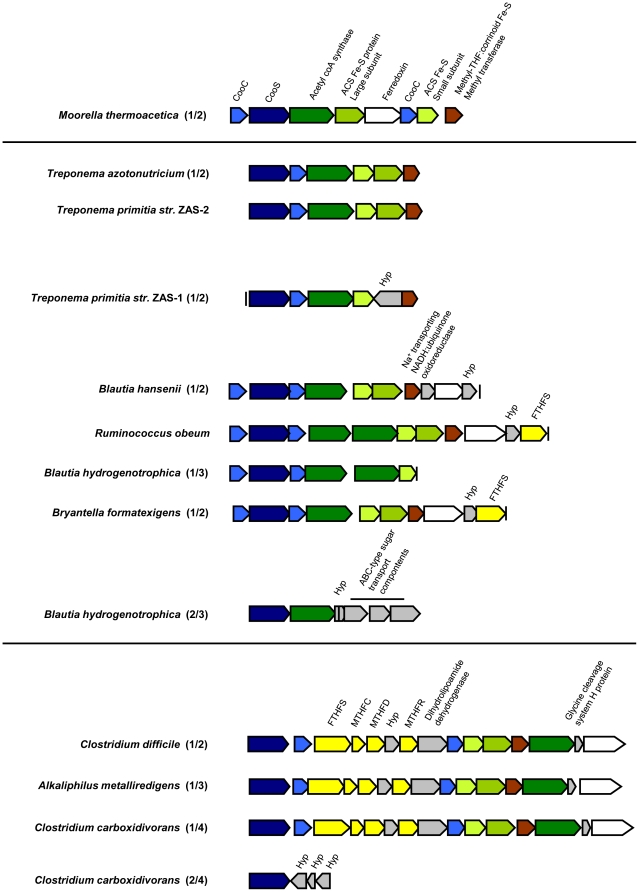
Gene neighborhood context of clade A-associated *cooS* genes. Genes adjacent to *cooS* were identified in sequenced or partially sequenced genomes of *T. azotonutricium*, *T. primitia* str. ZAS-1 and ZAS-2, and a *Nasutitermes* sp. metagenome and were compared to other species. Translated open reading frames (ORFs) were analyzed using BLAST [59] to compare the sequences to annotated proteins in the NCBI non-redundant sequence database. An expect (E) value cutoff score of <1e-5 to an annotated gene in the database was used to assign putative functions for ORFs given in the figure. ORFs not meeting this cutoff score are labeled as hypothetical (Hyp). Abbreviations given for ORFs encoding methyl branch enzymes of the acetyl-CoA pathway denote: formyl-tetrahydrofolate synthetase (FTHFS), methenyl-tetrahydrofolate cyclohydrolase (MTHFC), methylene-tetrahydrofolate dehydrogenase (MTHFD), and methylene-tetrahydrofolate reductase (MTHFR). Gene neighborhoods between horizontal lines contain *cooS* genes associated with clade A. Gene neighborhoods below contain *cooS* genes closely related to clade A (Fig. 2). The neighborhood for carbonyl branch genes of the acetyl-CoA pathway in *M. thermoacetica* is provided above for reference. Vertical bars indicate the end of a contig in partially sequenced genomes. For species having more than one *cooS* gene, the number out of the total is given parenthetically and corresponds to the numbering in figure 2.

In the model acetogen, *Moorella thermoacetica*, *cooS* and other genes involved in the carbonyl branch of the actyl-CoA pathway are located next to one another on the genome [15]. Analysis of *T. primitia* str. ZAS-1 and ZAS-2 and *T. azotonutricium* genome data showed that clade A-associated *cooS* genes are located near other genes for carbonyl-branch enzymes in each of these spirochetes (Fig. 6). Downstream from *cooS*, is a gene for the CODH accessory subunit (CooC), followed by acetyl-CoA synthetase (ACS), the ACS small and large Fe-S protein subunits, and a gene for a methyl-tetrahydrofolate corrinoid Fe-S methyl transferase, which delivers methyl groups generated in the methyl branch of the acetyl-CoA pathway to the ACS complex. While the gene order is not always conserved in clade A-associated *cooS* genes, most of the genes have a similar genome context. Some neighborhoods include genes for methyl-branch enzymes along with carbonyl branch enzymes of the acetyl-CoA pathway.

**Figure 7:**
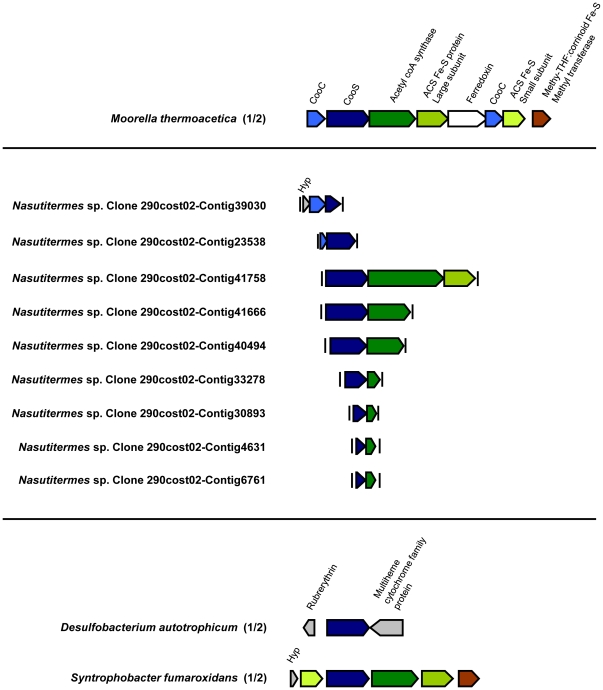
Gene neighborhood context of clade B-associated *cooS* genes. Genes adjacent to *cooS* were identified in a *Nasutitermes* sp. metagenome and were compared to other species. The genes were identified as described in Fig. 6 and labels and features of this figure are in accordance with that figure. Gene neighborhoods that contain *cooS* genes associated with clade B are located between the horizontal lines; those that are closely related to clade B (Fig. 2) are located below. The neighborhood for carbonyl branch genes of the acetyl-CoA pathway in *M. thermoacetica* is provided above for reference.

Much less genome context was available for *cooS* genes grouping within clade B, as there are no representative *cooS* genes of this clade in public databases of sequenced microbial genomes; however, two *cooS* genes and numerous short *cooS* gene fragments from the *Nasutitermes* sp. metagenome are associated with clade B (Fig. 7). The phylogenetic associations of *cooS* fragments were predicted by using a combination of a sliding window approach to reconstruct phylogenetic trees based on their translated amino acid sequences (which are not included in the construction of trees shown in figures) and by comparing their indels (as described above) to those found in full-length or near-full-length CooS sequences. Many of the *cooS* genes and gene fragments identified in the *Nasutitermes* metagenome as likely belonging to clade B are located adjacent to one or more genes or gene fragments predicted to encode other enzymes involved in the carbonyl branch of the acetyl-CoA pathway. Of the two CooS sequences that formed a moderately-supported (64% bootstrap support) sister clade to clade B (Fig. 2), the *cooS* gene from *Syntrophobacter fumaroxidans* is associated with other carbonyl-branch genes while the *cooS* gene from *Desulfobacterium autotrophicum* is associated with genes for a ruberythrin and a multi-heme cytochrome protein that are not part of the acetyl-CoA pathway.

**Figure 8:**
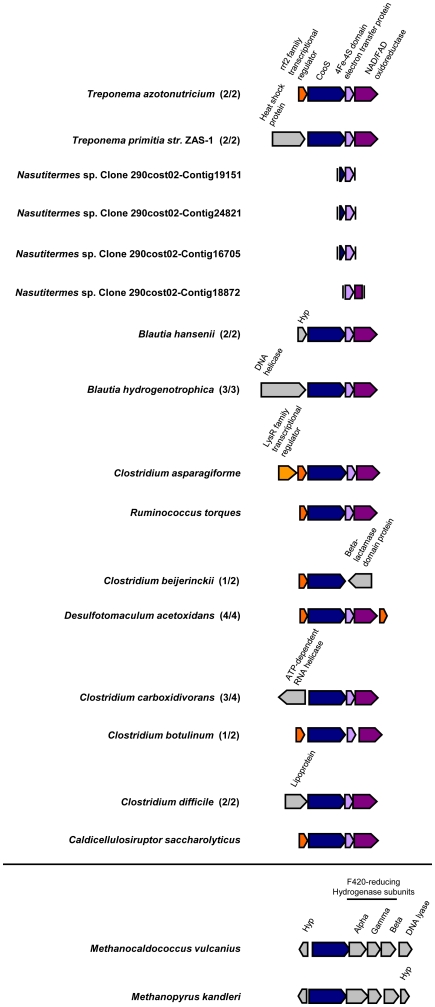
Gene neighborhood context of clade C-associated *cooS* genes. Genes adjacent to *cooS* were identified in sequenced or partially sequenced genomes of *T. azotonutricium*, *T. primitia* str. ZAS-1, and a *Nasutitermes* sp. metagenome and were compared to other species. The genes were identified as described in Fig. 6 and labels and features of this figure are in accordance with that figure. Gene neighborhoods that contain a *cooS* gene associated with clade C are located between the horizontal lines; those that are closely related to clade C (Fig. 2) are located below.

Clade C-associated *cooS* genes in *T. primitia* str. ZAS-1 and *T. azotonutricium* have a much different genome context than clade A and B-associated *cooS* genes. Unlike *cooS* genes associated with clade A or B, *cooS* genes associated with clade C from these spirochetes and other bacterial species are often found adjacent to a gene that encodes a 4Fe-4S domain electron transfer protein similar to the nitrate reductase electron transfer subunit NarB and a gene that encodes an FAD/NAD oxidoreductase, which has a high degree of amino acid sequence similarity to the nitrate reductase NADH oxidase subunit NarC (Fig. 8). The genome context of clade C-associated *cooS* genes thus resembles the Nar operon of clostridial species such as the characterized nitrate reductase of *Clostridium perfringens* [24]. While there is no sequence similarity between the catalytic subunit of nitrate reductase (NarA) and CODH enzymes, NarB and NarC from *C. perfringens* are homologous to the 4Fe-4S domain electron transfer protein (48% identity, 61% similarity) and the FAD/NAD oxidoreductase (37% identity, 60% similarity), respectively, of *T. azotonutricium*. Several bacterial *cooS* genes associated with clade C are preceded by a transcriptional regulator of the Rrf2 protein family (Pfam 02082) of helix-turn-helix domain DNA-binding proteins (Fig. 8). Proteins in this family of regulators have been shown to be repressors of genes involved in nitrite, nitric oxide, iron, and aromatic hydrocarbon metabolism [25], [26], [27], [28].

Several short CooS fragments from the *Nasutitermes* sp. metagenome group with clade C and are bioinformatically predicted to belong to species of *Treponema* [16]. They also show a gene arrangement predicted to be similar to other genes in this clade. By contrast, two CooS sequences from methanogenic *Archaea* (*Methanopyrus kandleri* and *Methanococcus vulcinus*), which form a well-supported out group to clade C (Fig. 2), are located next to genes for the alpha, beta, and gamma subunits of an F420-reducing hydrogenase (Fig. 8).

## Discussion

This report describes the first development and implementation of a degenerate primer set designed to amplify *cooS* genes, which encode the diverse catalytic (β) subunit of anaerobic carbon monoxide dehydrogenase (CODH) enzymes. These primers are distinct from other primers designed to amplify *coxL* genes, which encode aerobic carbon monoxide dehydrogenase enzymes [29]. While both anaerobic and aerobic CODH enzymes can catalyze the oxidation of CO to CO_2_, they are not homologous, have different subunit compositions, use different metal cofactors, and are found in microorganisms inhabiting vastly different environments.

Our inventories contain a broad diversity of *cooS* genes, each of which grouped into one of three distinct phylogenetic clades based on translated amino acid sequences. This pattern of diversity is recapitulated in each of the insect gut communities analyzed in the present study. The G+C content of *cooS* genes varied widely in each of the clades; however, genes for closely related enzymes within each clade often shared a comparable G+C content. Similar G+C content was also observed between *cooS* genes that belong to different clades but are from the same microbial species. This observation is consistent with the idea that CODH OTUs encoded by *cooS* genes of varying G+C content may come from a variety of bacterial species or genera. Therefore, our findings likely reflect the phylogeny of microbial species that utilize CODH enzymes as well as functional differences among CODH types used by microbes inhabiting the guts of termite and cockroach species.

It was not surprising to find that *cooS* genes from *Treponema* species are phylogenetically affiliated with those from several clostridial members of the phylum *Firmicutes*. Several of the genes for acetyl-CoA pathway in the termite gut treponeme *T. primitia* str. ZAS-2 have previously been shown to be most closely related to homologs found in members of the *Clostridia* [30], [31]. These findings have been hypothesized to imply that an ancient ancestor of extant termite treponemes acquired portions of the methyl branch of the acetyl-CoA pathway via lateral gene transfer from a clostridial species. As CooS sequences of clostridial origin are basal to genes of *Treponema* species both in clade A and clade C, the present study extends the hypothesis by suggesting that carbonyl branch genes of the acetyl-CoA pathway in termite gut treponemes may also have been acquired from a clostridial member of the phylum *Firmicutes* in the distant past.

Such evolutionary relationships among acetyl-CoA pathway genes complicate predictions about the phylogeny of bacteria that possess the various *cooS* genes identified in our inventories. Nevertheless, evidence based on phylogenetic inference that is consistent with the affiliation of protein sequences encoded by *cooS* genes from *Treponema* species suggests that group A1 (Fig. 3) and group C1 (Fig. 5) represent genes that belong to termite gut spirochetes of the genus *Treponema*. These groups contain many of the OTUs recovered from termite species and only a single OTU from a wood-feeding cockroach. An abundance and diversity of spirochetes in termite gut communities has long been recognized [16], [32], [33], [34] and our prediction that genes associated with group A1 and C1 are treponemal in origin is consistent with the results of a previous study that has documented similar variants binning to the genus *Treponema* via bioinformatic methods [16].

The large amount of CooS sequence divergence observed both in microbial species and in inventories collected in this study likely reflects functional differences among CODH types. That *cooS* genes associated with clades A, B, and C were present in each of the insects analyzed is significant and suggests that microorganisms employing these various CODH enzymes may occupy and benefit from a similar variety of niches in these related insect species, even though the gut microbial communities and physiologies as well as the insects' diets may differ.

*cooS* genes associated with clade A and B likely encode CODH enzymes used for acetogenic metabolism. Clade A includes representatives from each of the *Treponema* species isolated from the termite gut environment analyzed in this study, including *T. primitia* str. ZAS-1 and *T. primitia* str. ZAS-2, both of which have been shown to be CO_2_-reducing homoacetogens [35], [36]. The genome of *T. primitia* str. ZAS-2 has been closed and found to contain a single *cooS* gene belonging to clade A, meaning this gene is used for acetogenesis in this bacterial species. Gene neighborhood context for clade A and B-associated *cooS* genes in sequenced microbial genomes as well as the *Nasutitermes* sp. metagenome show that both of these genes are often located near other genes for carbonyl branch enzymes of the acetyl-CoA pathway. We therefore predict that clade A and B include enzymes that are directly involved in the acetyl-CoA pathway in the insects we analyzed. The phylogenetic relationship between these clades may reflect their similar function, as CooS sequences from clade A and B are more closely related than are those from either clade A and C or clade B and C. The prevalence of OTUs belonging to clade A and B in wood-feeding termites and the wood feeding cockroach where acetogenesis rates are high compared to the omnivorous cockroach [13], [14], [37] are consistent with these genes being associated with acetogenic metabolism. Curiously, clade A includes a CooS sequence from the termite gut isolate *T. azotonutricium*. This spirochete has never been shown capable of CO_2_-reductive acetogenesis [36] and repeated attempts have failed to reveal it capable of a relevant, cryptic physiology related to its possession of this gene. The role of this CODH enzyme in *T. azotonutricium* is an on-going area of research.

While clade A and B-associated *cooS* genes are likely used for acetogenesis, our analyses suggest that *cooS* genes associated with clade C may encode CODH enzymes used for metabolic functions that are not directly associated with this metabolism. First, bacteria possessing only *cooS* genes associated with clade C are not known to be capable of CO_2_-reductive acetogenesis [4]. Second, clade C CooS sequences are highly divergent from clade A and B sequences, possibly a result of selective pressure related to an alternative CODH function that may contribute to the sequence divergence. Finally, none of the *cooS* genes from clade C are located next to any other acetyl-CoA pathway gene on the genomes of sequenced microorganisms or *Nasutitermes* sp. metagenome fragments.

Both *T. primitia* str. ZAS-1 and *T. azotonutricium* possess one *cooS* gene associated with clade C and one associated with clade A, suggesting that both genes play a role in the physiology of certain termite gut spirochetes. As OTUs belonging to clade A and C together account for 90% of the total OTUs recovered in this study, these CODH enzymes likely play significant roles in the gut physiology of the insects we analyzed. There were, however, notable differences among insects in the distribution of CODH OTUs belonging to these clades. Our inventories contained a similar number of OTUs from clade A and clade C in all three termite species while the wood-feeding cockroach, *C. punctulatus*, inventory contained only a single OTU belonging to clade C. Conversely, the omnivorous cockroach, *P. americana*, inventory contained only 3 OTUs belonging to clade A while the overwhelming majority belonged to clade C. Although the distribution of CODH OTUs in cockroach species could be an artifact of primer bias, whereby our primers may preferentially amplify *cooS* genes from different clades in these insects, the result in the case of *P. americana* agrees with low rates of acetogenesis in omnivorous cockroaches and supports our prediction that clade C-associated CODH enzymes function in a metabolic capacity other than CO_2_-reductive acetogenesis.

Much of the research on anaerobic CODH has focused on several closely-related enzymes that are only distantly related to the variants discussed in this report. These include the CO-oxidizing CODH from *R. rubrum* [38], [39], [40] and *C. hydrogenoformans* [41], [42], [43], [44], the putative NADPH generating [42], [44] and acetyl-CoA-associated [45] CODH enzymes from *C. hydrogenoformans* and the acetyl-CoA-associated CODH from *M. thermoacetica* [46], [47], [48]. Studies have also found that certain CODH enzymes can catalyze other activities including 2,4,6-trinitrotoluene reduction, formate production, hydroxalamine reduction, and uptake hydrogenase activity either as naturally-occurring side reactions or as a result of protein engineering [49], [50], [51]. This enzymatic promiscuity may allow certain CODH enzymes to participate in other functions in some organisms.

Indeed, the genome of *C. hydrogenoformans* st. Z-2901 contains five genes (indicated as *C. hydrogenoformans* CODH-I–CODH-V on Fig. 2) for the catalytic subunit of CODH. The majority encode enzymes that group phylogenetically near other characterized enzymes with only CODH-V located near clade C. Several functions for these enzymes have been proposed based on gene neighborhood context or previous physiological investigations [52]. These include: 1) CODH-I, energy conservation via proton motive force generation using an associated proton-pumping hydrogenase; 2) CODH-II, NADPH generation; 3) CODH-III, carbon fixation via CO_2_-reductive acetogenesis; 4) CODH-IV, oxidative stress response via mitigation of reactive oxygen species in conjunction with a rubrerythrin-like protein; and 5) CODH-V, unknown function.

If clade C-associated CODH enzymes are not predicted to be directly involved in acetogenic metabolism, then what is their role in the physiology of the bacteria that possess them and why are they so prevalent in the guts of many of the insects investigated here? *cooS* genes associated with clade C are often located near a [4Fe-4S]-domain electron transfer protein and an NAD/FAD oxidoreductase. This is the case in *T. azotonutricium* and in *Clostridium ljungdahlii*. Both of these bacteria also have a clade A-associated CODH enzyme. The genome of *C. ljungdahlii* was recently sequenced [53] and the authors have proposed that one of the CODH enzymes (similar to the *T. azotonutricium* clade A enzyme) reduces CO_2_ through the carbonyl branch of the Acetyl-CoA pathway while the other (similar to the *T. azotonutricium* clade C enzyme) oxidizes CO and delivers the resulting CO_2_ to the methyl branch of the pathway. In support of their conclusions the authors showed that *C. ljungdahlii* can grow on CO+H_2_ in addition to CO_2_+H_2_, an ability likely afforded by the clade C-type CODH. This does not appear to be the case for *T. azotonutricium*. First, even with rigorous experimentation this bacterium has not been shown capable of CO_2_-reductive acetogenesis. The genome sequence of *T. azotonutricium* reveals that it lacks both a gene for formate dehydrogenase (which reduces CO_2_+H_2_ to formate and is the first enzyme in the methyl branch of Acetyl-CoA pathway) and a gene for methyl-THF reductase (an enzyme that generates a methyl group which is subsequently shuttled to the carbonyl branch of the pathway to form Acetyl-CoA). Second, *T. azotonutricium* does not appear to benefit from elevated levels of CO. A concentration of 1% CO in the culture headspace had an inhibitory effect on the growth of *T. azotonutricium* and lower levels provided no growth benefit (data not shown). The role of *cooS* genes belonging to clade C in termite gut bacteria is thus enigmatic but will be the subject of future investigations.

In addition to revealing new information about the phylogenetic and potential functional diversity of *cooS* genes utilized by members of the gut microbial communities of phylogenetically-lower, wood-feeding termites and related insects, this report provides a set of primers that can be used for molecular analysis of *cooS* diversity in other anaerobic microbial communities. We envision that these primers may be adapted to recover *cooS* genes from a variety of other environments where acetogenesis occurs [5]. This could allow interesting comparisons of anaerobic carbon monoxide dehydrogenase diversity and function to be drawn between free-living and host-associated environments. Finally, the modular design employed in the construction and application of these primers is a novel approach and may serve as a useful strategy to target genes of other sequence-diverse enzymes.

## Materials and Methods

### Primer design

Degenerate primer modules were designed to amplify *cooS* genes, which encode the catalytic (β) subunit of anaerobic carbon monoxide dehydrogenase (CODH) enzymes. These primers target nucleotide sequences corresponding to blocks of semi-conserved amino acids in an alignment of translated *cooS* genes found in sequenced bacterial genomes (GenBank) and *cooS* genes and gene fragments found in the metagenome of a *Nasutitermes sp.* hindgut community [16]. Additional details regarding the specific design and application of the primer modules are described below and in the results section. Sequences of primers are provided in Table 1.

### DNA templates

Genomic DNA samples from termite gut spirochete strains belonging to the genus *Treponema* and community DNA samples from the gut contents of various termites and cockroaches were extracted and purified. Isolated bacterial samples included previously described termite gut spirochete isolates *Treponema primitia* str. ZAS-1 and str. ZAS-2 and *T. azotonutricium* str. ZAS-9 [35], [36] and three previously isolated termite gut spirochetes, *Treponema* sp. ZAS-5, ZAS-6 and ZAS-8 [23], that are no longer viable in culture. DNA was extracted and purified from these bacteria using DNeasy Blood and Tissue Extraction kits according to the manufacturer's protocol for Gram-negative bacteria (QIAGEN, Valencia, CA). Insect specimens included gut contents from the worker castes of three lower termite species, *Zootermopsis nevadensis* (ChiA1), *Reticulitermes hesperus* (ChiA2), and *Insisitermes minor* (Pas1), and specimens of two cockroach species, *Cryptocercus punctulatus* (nymph and adult) and *Periplaneta americana* (adult). Insect gut community DNA was extracted using a combination of mechanical and chemical disruption according to the bead-beating/SDS/phenol procedure previously described [54] and purified using QIAGEN DNeasy columns as above. Specific details of these insect specimens have recently been reported [55].

### PCR amplification

Primer modules that produced amplicons of the expected length for *cooS* genes (approx. 1.4 kb) were determined for each of the samples (Fig. 1). Pair-wise amplifications using combinations of forward and reverse primer modules were carried out in 15 µl PCR volumes containing 1X FailSafe PCR PreMix D (Epicentre Biotechnologies, Madison, WI), 6 ng of DNA, forward and reverse primer modules at 0.2 pmol⋅degeneracy^−1^⋅primer^−1^ (final conc.), and 1U Taq DNA polymerase (New England Biolabs, Beverly, MA). Thermocycling conditions were as follows: 94°C for 2 min, followed by 30 cycles of 94°C for 30 sec, 55°C for 30 sec, and 72°C for 90 sec. The final extension was followed by an 8 min, 72°C run-off step.

To reduce non-specific amplification, PCR products for cloning were generated using only the forward and reverse primer modules that produced a correctly-sized amplicon. For each *Treponema* strain examined, a single 20 µl PCR was performed. For insect samples, PCRs were performed in triplicate. Each 20 µl PCR volume contained 1X FailSafe PCR PreMix D, 10 ng of DNA, 0.2 pmol⋅degeneracy^−1^⋅primer^−1^ (final conc.) of each primer module identified as productive in the initial pair-wise PCR screen and 1.75 U of Expand High-Fidelity Polymerase (Roche Diagnostics, Indianapolis, IN). Thermocycling conditions were as above, except that 25 cycles were used. After amplification, triplicate reactions were pooled for each insect sample.

### Library construction and sequencing

PCR products from *Treponema* samples were cloned directly as described below. Pooled PCR products from each insect sample were separated through 1% agarose preparatory gels. The 1.4 kb bands were excised from gels using a sterilized scalpel. DNAs contained in gel slices were extracted using QIAquick Gel Extraction Kits (QIAGEN) and eluted in 30 µl of nuclease-free dH_2_O. Adenosine overhangs were added back to the PCR products by performing end-repair reactions as follows: gel-purified PCR products (8 µl) were incubated for 15 min at 72°C in 10 µl reactions containing 1X ThermoPol Buffer, 1 mM dNTP and 1U Taq DNA polymerase (New England Biolabs, Beverly, MA).

PCR products from *Treponema* isolates and gel-purified PCR products from insect gut communities were cloned into pCR4-TOPO vectors at room temperature for 30 min using a TOPO TA Cloning Kit (Invitrogen, Carlsbad, CA). One Shot TOP 10 chemically competent cells (Invitrogen) were transformed with cloning reactions and were grown overnight on LB agar plates containing ampicillin (100 µg/ml) for selection. Colonies were selected after growth and sub-cloned on LB agar with selection as above. Clone insert size was verified by colony PCR using vector-specific primers T3 and T7 (Invitrogen). Clones from several insect gut communites (*Z. nevadensis*, *C. punctulatus* nymph, and *P. americana*) were additionally screened for restriction-length polymorphisms (RFLP) after digestion with 0.3 U⋅µl^−1^ of HinPI (New England Biolabs) at 37°C for 4 hours and subsequent gel-electrophoresis through 2.5% agarose gels.

Colonies selected for sequencing were grown overnight at 37°C in LB liquid cultures under ampicillin selection (100 µg/ml). Plasmids were extracted and purified using QIAprep Miniprep Spin columns (QIAGEN) according to the manufacturer's protocol. Plasmid insert DNA was sequenced by Laragen, Inc. (Los Angeles, CA) using automated Sanger sequencing and vector-specific primers T3 and T7 (Invitrogen). Sequences were assembled and edited using the SeqMan module of the Lasergene software package (DNASTAR Inc, Madison, WI). Translated amino acid sequences were aligned using ClustalW [56] and were grouped by operational taxonomic units based on a 97% amino acid similarity cutoff. Inventory diversity (Table S1) was estimated using the software program EstimateS (Version 8.2, R. K. Colwell, http://purl.oclc.org/estimates).

### Phylogenetic analysis

Phylogenetic analyses were performed using the ARB software package v. 05.05.26 [57]. Protein phylogenies and bootstrap values were determined by Phylip protein maximum likelihood methods using aligned datasets and conservative filters that disregarded columns of data containing sequence gaps. Specific details of tree reconstruction for each phylogenetic tree are given in the figure legends.

### Genome sequencing and sequence availability

The details of the genome sequencing and closure of *T. azotonutricium* ZAS-9 (Genbank CP001841) and *T. primitia* ZAS-2 (Genbank CP001843) will be reported elsewhere in full (S. Tetu, X. Zhang, A. Rosenthal, Q. Ren, R. Seshadri, E. Matson, L. Elbourne, K. Hassan, A. Durkin, D. Radune, Y. Mohamoud, R. Shay, S. Jin, K. Lucey, N. Ballor, E. Ottesen, A. Allen, J. Leadbetter, and I. Paulsen. (*in preparation*). A draft sequence of *Treponema primitia* strain ZAS-1 (GenBank AEEA00000000) was obtained via 454 pyrosequencing by Richard White and Stephen Quake at Stanford University, and was annotated using RAST [58]. All *cooS* sequences identified in the present study have been submitted individually to the GenBank and EMBL databases under accession numbers: HQ900690–HQ900840.

## Supporting Information

Figure S1 ***cooS*** **amplification products from termite gut communities.** 4-µl samples of PCR products generated by combined primer modules cooS-2F, cooS-4F, cooS-1R and cooS-2R from each termite template were run on a 1.5% agarose gel and visualized by staining with ethidium bromide. Gel purification of the 1.4 kb bands from separate preparatory gels was performed to remove non-specific amplification products and non-reacted primers. PCR reaction conditions are described in methods. Control reaction received no template.
(TIF)

Figure S2 **Collector's curves of RFLP types recovered in three insect libraries.** The frequency of recovery is given as the number of counts for each RFLP type identified in the libraries: panel A, *Z. nevadensis*; panel B, *C. punctulatus* nymph; panel C, *P. americana*. Black bars, RFLPs that were sequenced and confirmed as *cooS*; gray bars, RFLPs that were sequenced and found not to be *cooS*; open bars, RFLPs that were sequenced and contained mixed sequencing signals.
(TIF)

Table S1 Details of insect gut inventory construction and analysis.
(DOC)
