# Supplementary figures and images for: Anaerobic Carbon Monoxide Dehydrogenase Diversity in the Homoacetogenic Hindgut Microbial Communities of Lower Termites and the Wood Roach
Source: PLoS One. 2011 Apr 26;6(4):e19316. doi: 10.1371/journal.pone.0019316 (PMC3082573; doi:10.1371/journal.pone.0019316)

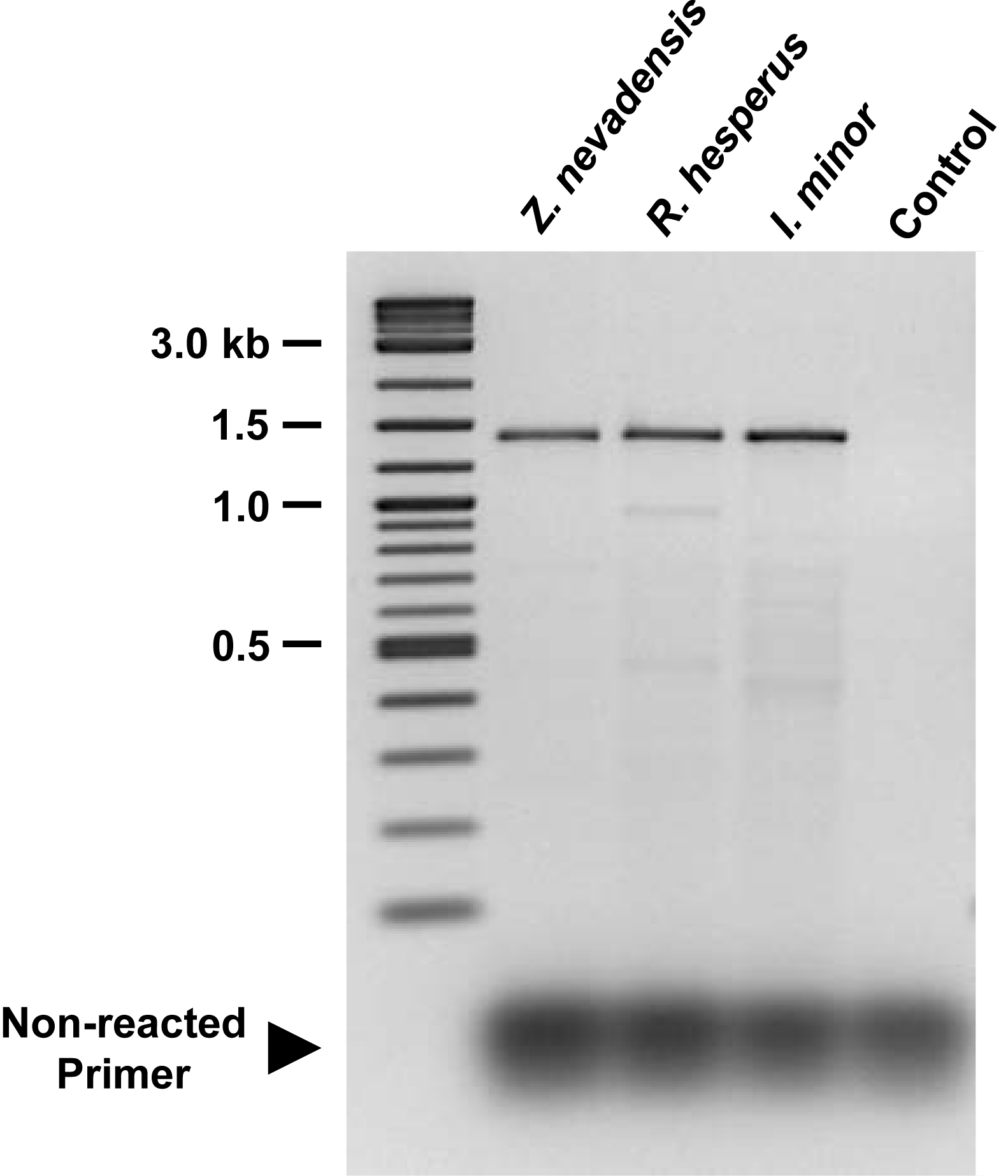

Supplement: Figure S1 — cooS amplification products from termite gut communities. 4-µl samples of PCR products generated by combined primer modules cooS-2F, cooS-4F, cooS-1R and cooS-2R from each termite template were run on a 1.5% agarose gel and visualized by staining with ethidium bromide. Gel purification of the 1.4 kb bands from separate preparatory gels was performed to remove non-specific amplification products and non-reacted primers. PCR reaction conditions are described in methods. Control reaction received no template. (TIF) [file pone.0019316.s001.tif]

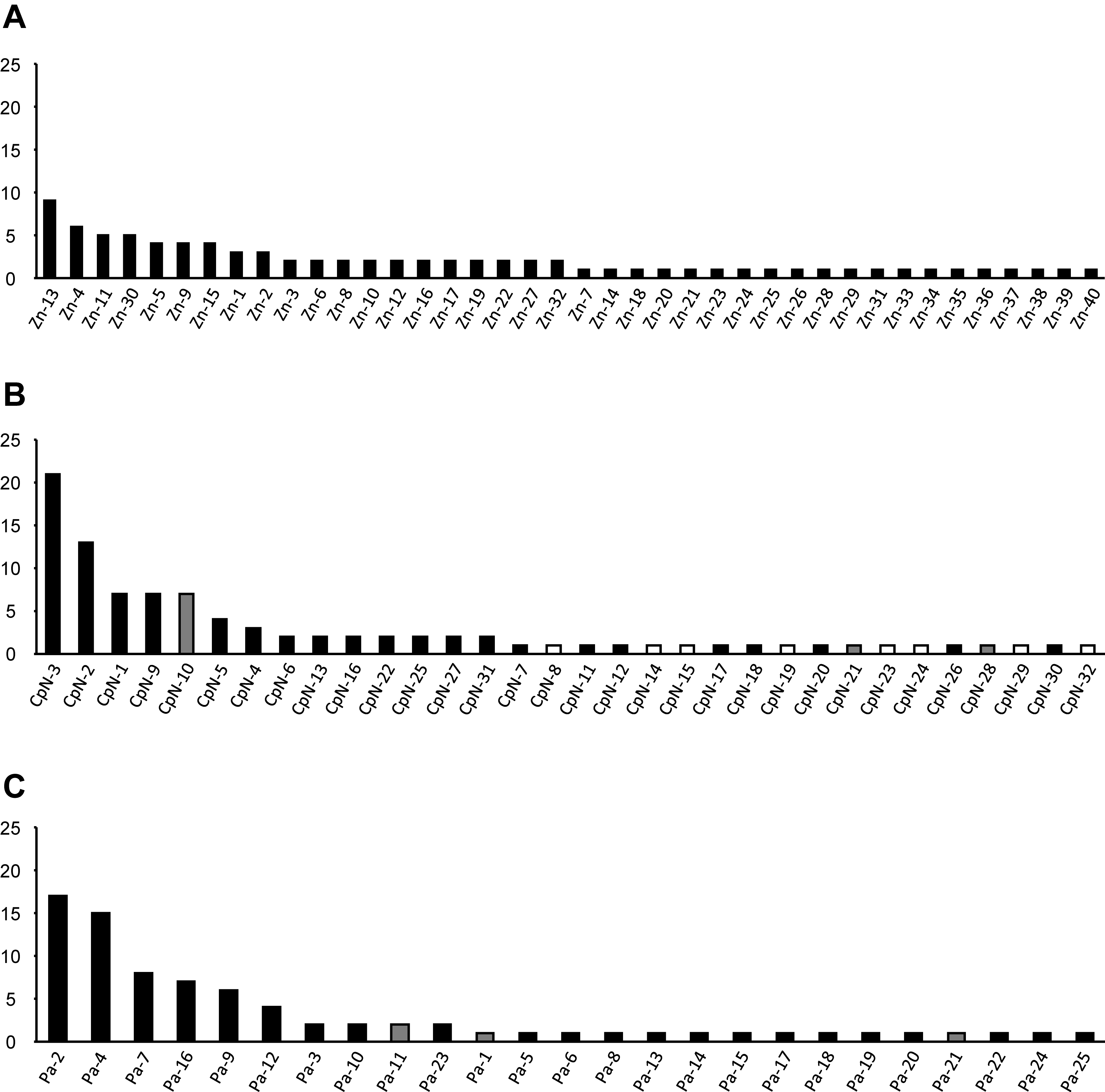

Supplement: Figure S2 — Collector's curves of RFLP types recovered in three insect libraries. The frequency of recovery is given as the number of counts for each RFLP type identified in the libraries: panel A, Z. nevadensis; panel B, C. punctulatus nymph; panel C, P. americana. Black bars, RFLPs that were sequenced and confirmed as cooS; gray bars, RFLPs that were sequenced and found not to be cooS; open bars, RFLPs that were sequenced and contained mixed sequencing signals. (TIF) [file pone.0019316.s002.tif]
