# Supplementary material for: Anaerobic Carbon Monoxide Dehydrogenase Diversity in the Homoacetogenic Hindgut Microbial Communities of Lower Termites and the Wood Roach
Source: PLoS One. 2011 Apr 26;6(4):e19316. doi: 10.1371/journal.pone.0019316 (PMC3082573; doi:10.1371/journal.pone.0019316)
Supplement: Table S1 — Details of insect gut inventory construction and analysis. (DOC) [file pone.0019316.s003.doc]

|  |  |  |  |  |  |  | |  |  |
| --- | --- | --- | --- | --- | --- | --- | --- | --- | --- |
|  | **No. Clones** | **Correct Insert Size^1^** | **No. RFLP Types** | **SeqencedClones** | ***cooS* Sequences^2^** | **CODHOTUs^3^** | | **Mean Chao1 (SD)**^4^ | **95% LCI, HCI**^5^ |
| **Insect Gut Inventories** |
|  |  |  |  |  |  |  | |  |  |
|  |  |  |  |  |  |  | |  |  |
| ***Zootermopsis nevadensis* (worker)** | 95 | 85 | 40 | 40 | 40 | **27** | | 25.3 (4.1) | 22.2, 42.8 |
| ***Reticulitermes hesperus* (worker)** | 24 | N/A | N/A | 24 | 24 | **13** | | N/A | N/A |
| ***Incisitermes minor* (worker)** | 24 | N/A | N/A | 24 | 24 | **9** | | N/A | N/A |
| ***Cryptocercus punctulatus* (nymph)** | 95 | 94 | 32 | 32 | 21 | **10** | | 9.4 (1.7) | 8.5, 17.6 |
| ***Cryptocercus punctulatus* (adult)** | 24 | N/A | N/A | 24 | 14 | **7** | | N/A | N/A |
| ***Periplaneta americana* (adult)** | 95 | 80 | 25 | 25 | 22 | **19** | | 18.7 (3.9) | 15.9, 36.3 |
|  |  |  |  |  |  |  | |  |  |
| ^1^ Based on an amplicon size of approx. 1.4 kb | | | | | | |  | | |
| ^2^ Based on BLAST analysis of sequenced inserts (some clones contained non-target DNA) | | | | | | |  | | |
| ^3^ Based on a 97% amino acid similarity cutoff | | | | | | |  | | |
| ^4^ Mean of the diversity estimator Chao1 (SD, standard deviation) calculated using EstimateS (Colwell, 2009) | | | | | | |  | | |
| ^5^ Lower (LCI) and higher (HCI) 95% confidence intervals for mean Chao1 | | | | | | |  | | |
| N/A, not analyzed | | | | | | |  | | |
